# Who was buried with Nestor’s Cup? Macroscopic and microscopic analyses of the cremated remains from Tomb 168 (second half of the 8^th^ century BCE, Pithekoussai, Ischia Island, Italy)

**DOI:** 10.1371/journal.pone.0257368

**Published:** 2021-10-06

**Authors:** Melania Gigante, Alessia Nava, Robert R. Paine, Ivana Fiore, Francesca Alhaique, Carmen Mariarosaria Esposito, Alessandra Sperduti, Jacopo Bonetto, Teresa Elena Cinquantaquattro, Bruno d’Agostino, Luca Bondioli

**Affiliations:** 1 Department of Cultural Heritage, University of Padua, Padua, Italy; 2 DANTE-Diet and Ancient Technology Laboratory, Department of Maxilla-Facial Sciences, Sapienza University of Rome, Rome, Italy; 3 Skeletal Biology Research Centre, School of Anthropology and Conservation, University of Kent, Canterbury, United Kingdom; 4 Department of Environmental Biology, Sapienza University of Rome, Rome, Italy; 5 Bioarchaeology Service, Museum of Civilizations, Rome, Italy; 6 School of Natural and Built Environment, Queen’s University Belfast, Belfast, United Kingdom; 7 Department of Asia Africa and Mediterranean, University of Naples ‘L’ Orientale’, Naples, Italy; 8 Soprintendenza Archeologia, Belle Arti e Paesaggio per l’area metropolitana di Napoli, Naples, Italy; 9 Department of Cultural Heritage, University of Bologna, Alma Mater Studiorum, Ravenna, Italy; Institute for Anthropological Research, CROATIA

## Abstract

Cremation 168 from the second half of the 8^th^ century BCE (Pithekoussai’s necropolis, Ischia Island, Italy), better known as the Tomb of Nestor’s Cup, is widely considered as one of the most intriguing discoveries in the Mediterranean Pre-Classic archaeology. A drinking cup, from which the Tomb’s name derives, bears one of the earliest surviving examples of written Greek, representing the oldest Homeric poetry ever recovered. According to previous osteological analyses, the Cup is associated with the cremated remains of a juvenile, aged approximately 10–14 years at death. Since then, a vast body of literature has attempted to explain the unique association between the exceptionality of the grave good complex, the symposiac and erotic evocation of the Nestor’s Cup inscription with the young age of the individual buried with it. This paper reconsiders previous assessments of the remains by combining gross morphology with qualitative histology and histomorphometric analyses of the burnt bone fragments. This work reveals the commingled nature of the bone assemblage, identifying for the first time, more than one human individual mixed with faunal remains. These outcomes dramatically change previous reconstructions of the cremation deposit, rewriting the answer to the question: who was buried with Nestor’s Cup?.

## Introduction

**Fig 1:**
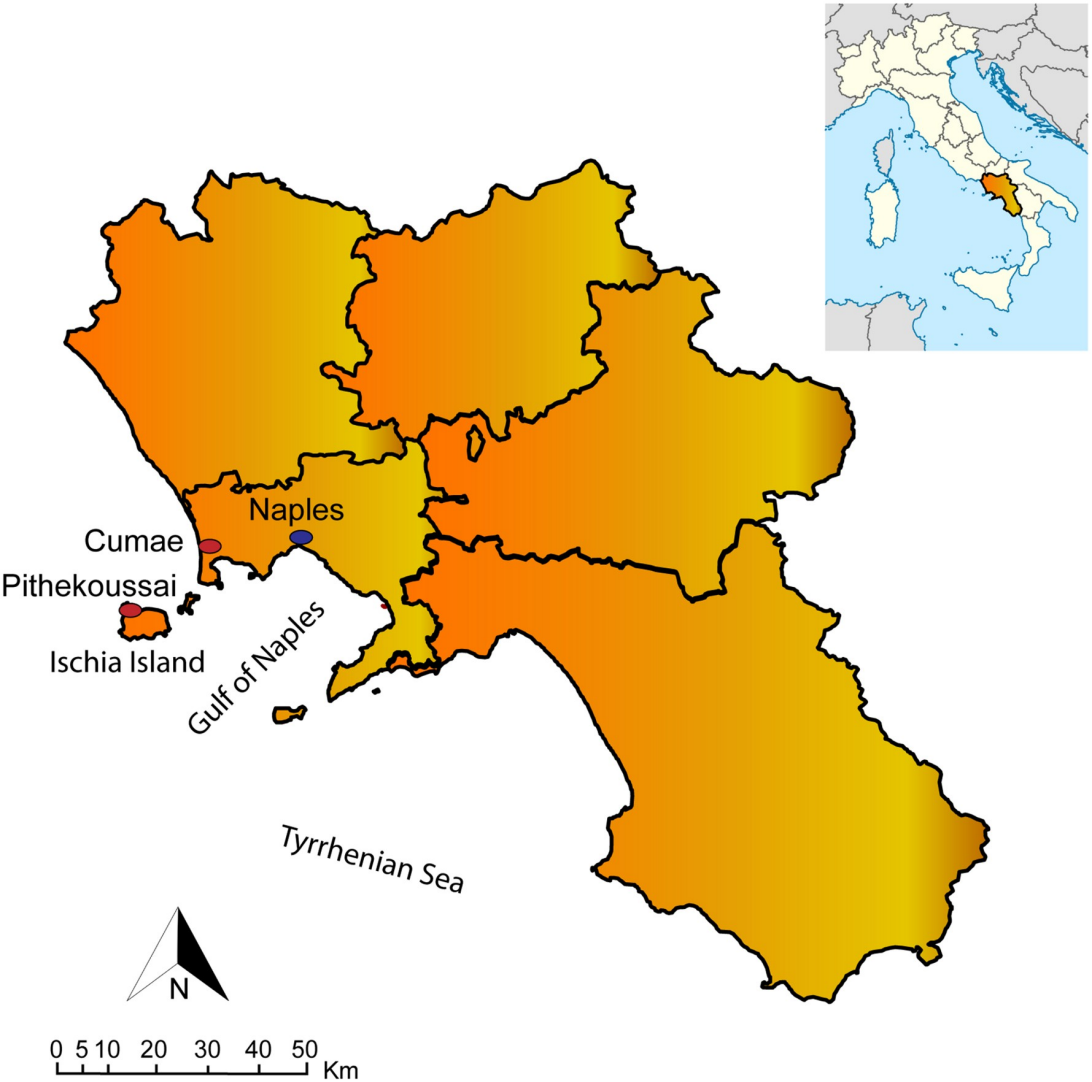
Pithekoussai, current Ischia Island, the major Island of the Phlegrean district, in the Gulf of Naples, in the Campania region (South Italy). On the mainland, the cities of Naples and Cumae.

The Pithekoussai ancient settlement and necropolis are situated on Ischia Island, in the Bay of Naples, Italy (Fig 1). The site is the earliest Greek outpost in the West Mediterranean [1].

Pithekoussai ancient settlement was founded by Greeks from Euboea, the large island east of the Attic Peninsula, in the 8^th^ century BCE like Cumae, a city located a short distance from Naples, in South Italy (Strabo, *Geographia*, V, 4, 9; Livy, *Ab Urbe Condita*, VIII, 22). A substantial portion of published research for Pithekoussai has focused on identifying the role the site played in the early stages of the Iron Age Greek colonization of the West. Ancient written sources and archaeological evidence suggest that Pithekoussai was an emporium, favouring encounters between Phoenicians and Greeks, the first impulse for the spread of alphabetic writing, the knowledge of Homeric poems, and the circulation of luxury goods, manners and customs from the Near East.

**Fig 2:**
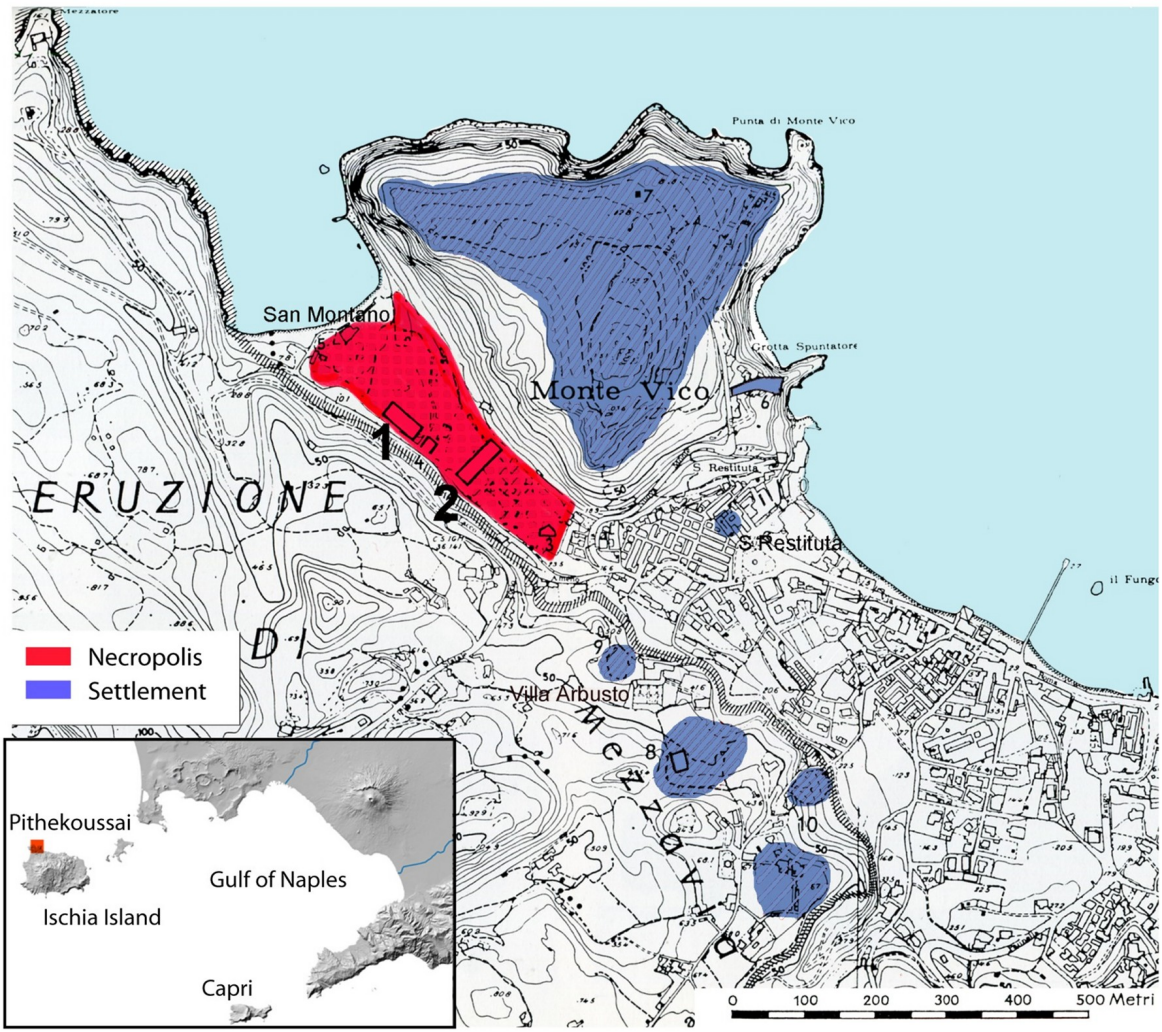
The location of the Pithekoussai necropolis and settlement on the Ischia Island (elaborated from [2], plan 2, by T.E. Cinquantaquattro). The geographical location of the Pithekoussai necropolis and settlement on the Ischia Island, Gulf of Naples (Campania, South of Italy). In red, the two different batches of the necropolis excavations: (1) *Pithekoussai I* (Buchner’s excavations 1952–1961); (2) *Pithekoussai II* (Buchner’s excavations 1965–1982). In blue, the settlement areas, located in the present-day town of Lacco Ameno, on Monte Vico, and the surrounding hills.

The necropolis was brought to light by G. Buchner, who excavated 1300 tombs over thirty years (1952–1982), many of which have been published [2–4]. Excavations revealed that the necropolis extended across San Montano’s valley, on the northwestern corner of the Ischia Island (Fig 2).

The majority of tombs dated to the 8^th^ -7^th^ century BCE constitute formidable evidence for knowledge of the society of the time. Three funerary practices have been identified in the necropolis [2]. Inhumation is the most common ritual regardless of age-at-death. This funerary custom includes inhumations in pit grave for sub-adults and adults, and *enchytrismos*, namely inhumation in amphora, for newborns, more rarely for infants [5]. Conversely, secondary cremations, placed in a shallow circular pit and covered with a stone tumulus [2], are usually designated to the adult population but there are some exceptions to this practice [5].

**Fig 3:**
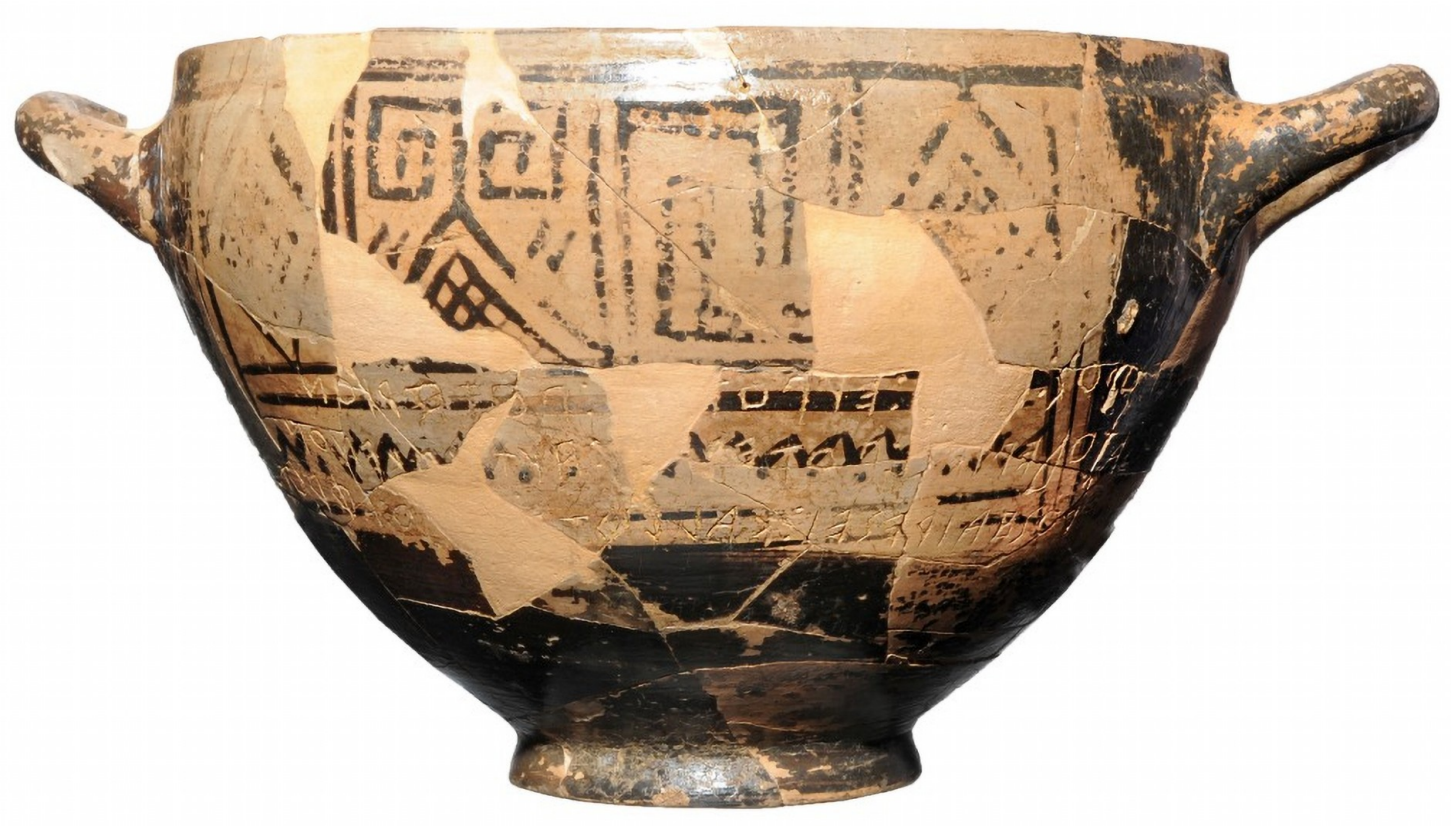
Nestor’s Cup from Cremation 168, Pithekoussai’s necropolis. The Cup is on permanent display at the Museo Archeologico Nazionale di Villa Arbusto, Lacco Ameno (Ischia Island). The metric inscription, partially in hexameter verses, translates roughly to ***’****I am Nestor’s cup*, *good to drink from*. *Whoever drinks this cup empty*, *straightaway desire for beautiful-crowned Aphrodite will seize him****’*** (picture from *Soprintendenza Archeologia*, *Belle Arti e Paesaggio per l****’****area metropolitana di Napoli*).

Tomb 168 is a cremation dated to the second half of the 8^th^ century BCE. This tomb is better known as the Tomb of Nestor’s Cup and represents one of the most significant findings from the Pithekoussai necropolis [2,6,7]. The tomb is named after an outstanding grave good (Nestor’s Cup) which has captured archaeologists’ interest (e.g. [8–11]). Nestor’s Cup is a *kotyle* from northern Ionia (Asia Minor) [12], bearing one of the earliest known Greek inscriptions (Fig 3). Traced very accurately in the Euboean alphabet, the metric inscription draws on a *topos* of Greek poetry associating symposiac and erotic practices and references the Homeric epic, specifically the mention of Nestor’s famous Cup in the Iliad. However, the identification of the *kotyle* as Nestor’s renowned Cup is paradoxical since it is a modest clay vessel used to drink wine. In contrast, Nestor’s Cup evoked by Homer (Iliad, 11.632–637) was ‘*a beautiful cup… studded with golden nails; on each handle*, *a pair of golden doves was fed’*, and it was so big that only Nestor could lift it from the table when it was full. The cup was used to drink a thick fortifying beverage reserved for heroes.

Nestor’s Cup found at Pithekoussai is placed at the centre of a vast literature [8–11,13], being a highly significant chronological marker for specific aspects of language, epigraphy, archaeology, history, and literature. The dating of the context (S1 Text in S1 File) and the osteological analysis of the skeletal remains buried with the Cup have important implications for the several disciplines involved.

In the last few years, interest in cremated remains has increased in bioarchaeological fields, allowing for the development of a range of more sophisticated techniques to investigate past cremation practices and the biological profiles of burnt skeletons (e.g., [14–19]). Even though several studies have discussed the alterations of bone and dental microstructure as a function of incremental heating (e.g., [20–29]), others have focused on the use of the histological and histomorphometric analysis of cremated specimens for various purposes (e.g., [14,30–34]). Despite the heat-related changes to bone microstructure, the use of histological analysis of cremated remains to distinguish between human and non-human bones has proven to be adequate (e.g., [14]).

Research concerning the histological structure of bone tissues has been successfully employed in forensic anthropology and archaeology for several goals, including for distinguishing between human and non-human bone when chemical investigations could not be employed (e.g., [14–26]). Histologically, bone structure is characterized by different micro-architectures amongst different animals. Comparative studies have demonstrated mainly how the presence versus absence of plexiform bone and osteon banding, i.e., a linear organization of the secondary osteons and the size of the Haversian system and bone structures’ geometry, could be helpful information regarding histological and histomorphometric interspecies variations. Mulhern and Ubelaker [23] described that the osteon banding organization is typically attested in sheep, impala, equids, and non-human primates but not in adult humans (*also in* Nor *et al*.) [28]. Plexiform bone is commonly attested in cow and pig femora [29] but not in human bones. Traditionally, interspecies variabilities have been detected considering some parameters of the Haversian system, such as the Secondary Osteon Area (On.Ar.) and Haversian Canal Area (Hr.Ar.) (S1 Table) [19,30–34]. Intraspecies variability amongst humans has also been histologically explored to understand the remodelling activities of the secondary structures of bone tissues related to age, biomechanics, and endocrine factors (e.g., [35–38]). Comparative studies in humans of known age-at-death, sex, and clinical history have been successfully employed to elaborate age prediction models applied to modern, ancient and fossil bones (e.g., [39–43]).

### The case study of the Tomb of Nestor’s Cup. Buchner’s hypotheses and inherited problems

The excavation of the Tomb of Nestor’s Cup has posed several interpretive issues since its tumulus (burial mound) had been entirely demolished by subsequent graves. Buchner did not conceal his doubts about how the burial complex should have been interpreted [2,10,13]. The difficulty of the task was further compounded because the investigation was carried out in two separate seasons, in October 1954 and in June 1955. In the first season, as we gather from the excavation journal, Buchner designated as Cremation 168 a large ***‘***lens of black earth***’***, a layer (3.80x2 m) containing ashes and bones collected from the funeral pyre along with the grave goods. The excavation of the ***‘***lens of black earth***’*** revealed three depressions at its bottom, a circumstance which led Buchner, in the first instance, to the hypothesis that they belonged to three originally distinct cremations. He specified that when he dug the ***‘***lens of black earth***’*** it was impossible to distinguish the materials by their disposition, and moreover, Nestor’s Cup fragments were scattered across the ***‘***lens of black earth***’*** [13].

**Fig 4:**
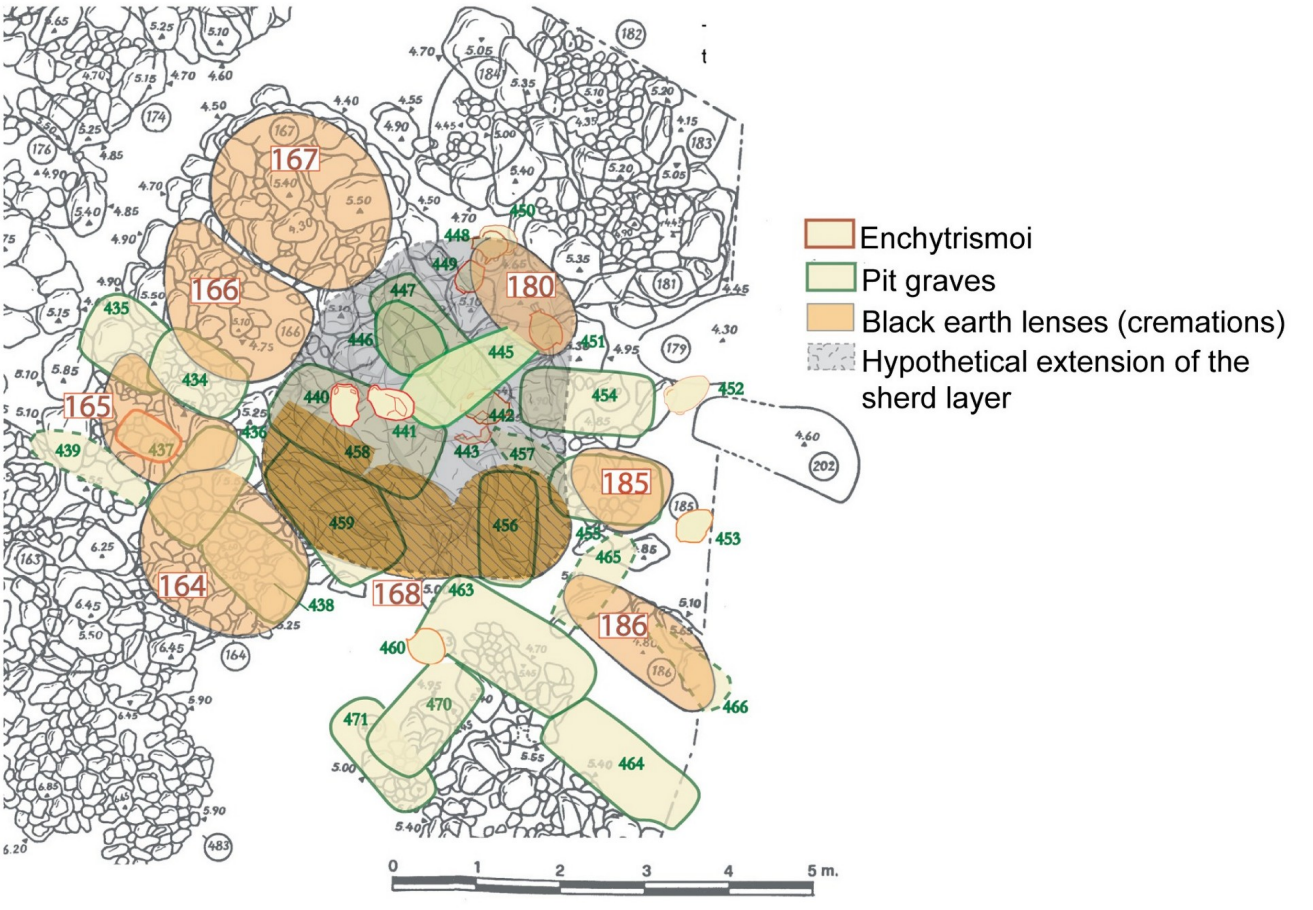
The Tomb of Nestor’s Cup (Cremation 168) and the cremation tumuli near it (elaborated from [2], plan AII and AII *bis* by T.E. Cinquantaquattro). The cluster of Cremation 168 and Tumuli 164, 165, 166, 167, 180, 185, 186 (from left to right). In dark yellow, the ***’***black earth lenses***’*** and the three depressions of Cremation 168. In grey, the ***’***sherd layer***’*** of Cremation 168.

In 1955 Buchner investigated the relations between Tomb 168 and the nearby tumuli (Fig 4).

In a layer ‘of abundant ***‘***burnt sherds mixed with brown earth***’*** (4x3 m) extending northward between tumuli 166–167 and 180, and southward underneath Tomb 168, he recovered sherds that joined others from the ***‘***lens of black earth’. Importantly, near Tumuli 166 and 167, Buchner found three sherds of the inscribed Cup and one of a crater. The crater is a type of vase uncommon in Pithekoussai’s tombs and it exhibits a painted inscription [13]. This ***‘***sherd layer***’*** yielded at least 24 vases, including *skyphoi*, *kotylai*, *kyathoi*, *lekythoi* and *aryballoi* [2]. We should also consider the possibility that, although subsequent in time, the formation of the ***‘***sherd layer***’*** and that of the overlaying ***‘***lens of black earth***’*** could be the results of two distinct actions in the same funeral ceremony, the dissemination of the products of the pyre and the deposition of the ashes, respectively. The whole was then supposedly jumbled by subsequent disturbances.

Alternatively in the final edition of his excavation, Buchner interpreted the context as a single burial, albeit being a heavily disturbed one [2]. The decisive argument supporting the idea of a single cremation resulted from the preliminary osteological examinations of the cremated remains. T.F. Spence and then M.J. Becker agreed on a single individual’s presence, assessing a different age-at-death. Spence identified an individual aged ten years at death, whereas Becker recognized an individual between 12 and 14 years [2,44,45]. No faunal remains were identified or reported from the Tomb.

The significant number of grave goods, the inscribed *kotyle*, the presence of four craters (one of them yielded the painted inscription ***‘****ex theo****’*** on the stem of the foot and interpreted as a *sakrale Inschrift* [7,10]), and the attribution of the cremation deposit to a juvenile–an exception to the funerary customs at Pithekoussai–highlighted the extraordinary character of the Tomb and caught scholars’ interest [7]. Indeed, one of the most debated issues concerned the unique association of the outstanding material culture assemblage and the symposiac and erotic allusion to the Homeric poetry of Nestor’s inscription with the young age-at-death of the individual buried with it.

Due to the importance of the context within the Mediterranean Pre-Classic archaeology, the conflicting results and divergent interpretations of the Tomb and its findings, this paper re-examines the cremated remains from a new and more exhaustive perspective. The study couples traditional and advanced osteological methods to shed more light on this puzzling funerary context, opening to a deeper and more comprehensive understanding of both the bone assemblage and funerary practices.

## Results

### Preliminary gross morphological and osteometric results

The burned skeletal assemblage from the Tomb of Nestor’s Cup consists of 195 fragments. The cremated remains show heterogeneous dimensions ranging from <1 cm to ~14 cm. The total weight of the remains is 218.4 g. All pieces are characterized by profound heat-induced alterations, including fractures and modifications in size and shape (e.g., warping, delamination, fissures, and shrinkage) [46–49]. The remains show a chromatic variability from brownish-blackish to calcined white [47,50]. The detailed scoring of the cremated remains allowed for the morphological identification of both human and non-human bones. Table 1 shows weights and percentages in weight of the bone fragments, subdivided into three diagnostic categories based on macroscopic observations: (i) human skeletal remains; (ii) non-human skeletal remains; (iii) undetermined skeletal remains. The last category includes tiny fragments for which taxonomic attribution by the gross anatomical investigation is doubtful or not possible to perform.

**Table 1:** Composition of the cremated remains from the Tomb of Nestor’s Cup.

	Weight (g)	Percentage in weight (%)
**Human skeletal remains**	166.1	76
**Non-human skeletal remains**	39.2	18
**UND**	13.1	6
**Total**	**218.4**	**100**

Weights and percentages of the bone fragments for three diagnostic categories based on gross morphology: (i) human skeletal remains; (ii) non-human skeletal remains; (iii) UND = undetermined skeletal remains.

### The human sub-sample

**Fig 5:**
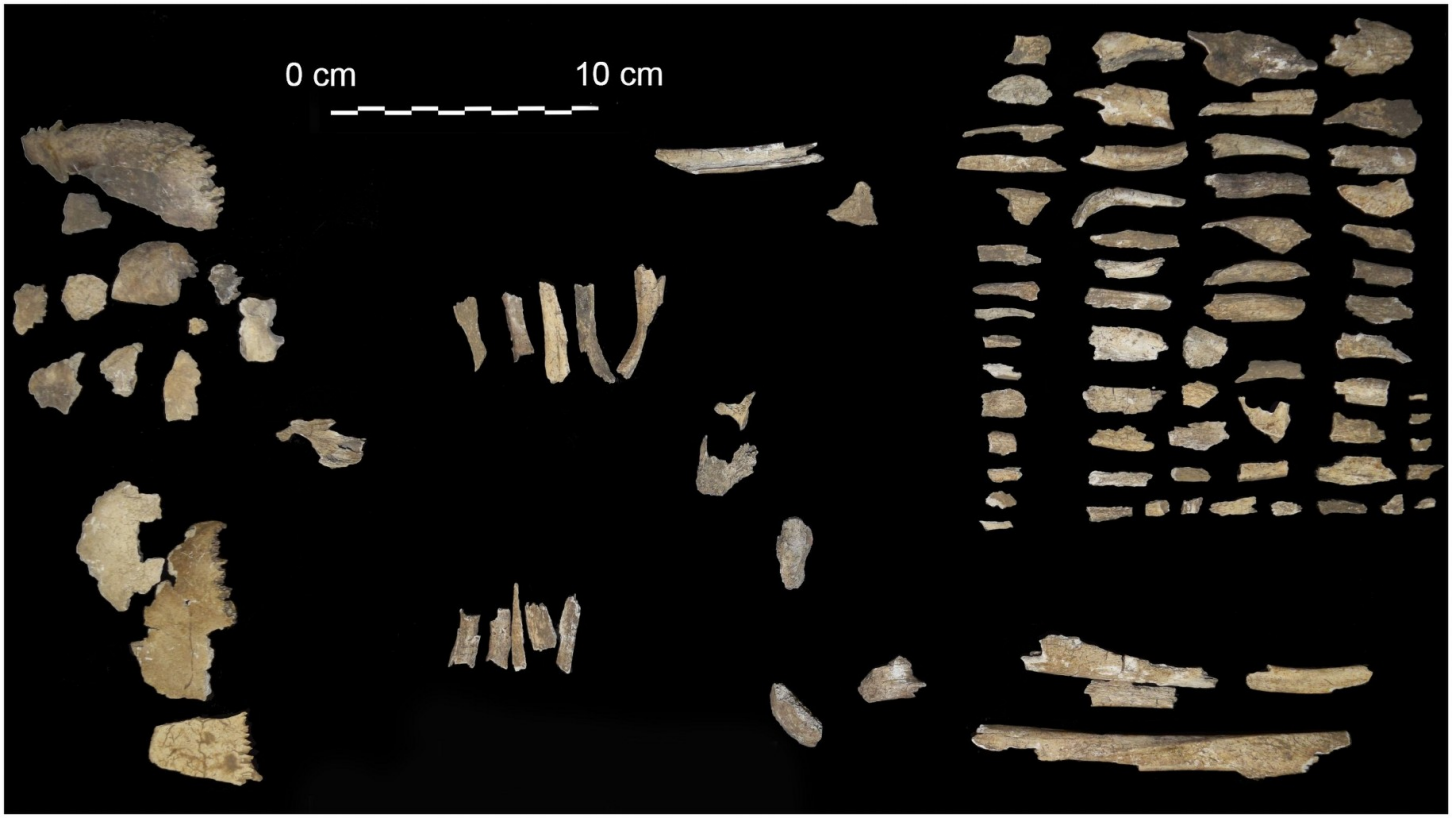
The human fragments from the Tomb of Nestor’s Cup. Bone and dental fragments recognized as human by the gross morphology observations.

Remains macroscopically recognized as human consists of 130 fragments, corresponding to a total weight of 166.1 g (76% of the total sample) (Fig 5 and Table 2).

**Table 2:** Composition of the human cremated assemblage from the Tomb of Nestor’s Cup.

	No. fragments	Weights (g)	Percentage in weight (%)
**Cranium**	15	45.5	27
**Axis and pelvis**	9	6.4	4
**Large Ø bone**	103	111.05	67
**Small Ø bone**	3	3.15	2
**Total**	**130**	**166.1**	**100**

The number of fragments, weights, and percentage in weight of human cremated remains per main anatomical categories.

Due to the absence of more specific morphological traits, the remains were divided into broad anatomical categories: (i) cranium; (ii) axis and pelvis; (iii) large-diameter long bones (humerus, femur and tibia); (iv) small-diameter long bones (radius, ulna, and fibula). Teeth are absent, except for a small fragment of one dental root, possibly attributed to a permanent molar. Table 2 reports the number, weight, and percentage in weight of the human bone assemblage, subdivided by gross anatomical categories.

Human bone fragments show prevalent grey and white chromatic changes of the cortical surfaces (85%), while a minority of specimens shows a black colour (15%). Cranial fragments are characterized by a chromatic transition from grey-bluish to whitish-calcinate white. The latter is predominant. Charred discolourations are observed in some long bone fragments, though most postcranial bones are lighter in colour. The presence of transverse fracturing and extensive warping of cranial bones and some long bones suggests that the cremation ritual did not involve dried, fat-free, bones [47,51]. Following Symes *et al*. [47] and Walker *et al*. [50], the cremated remains burning temperature was between 600°C and 900°C. The high degree of fragmentation and the absence of diagnostic bone fragments hamper the Minimum Number of Individuals (MNI) estimation based on the macroscopic observation. Nevertheless, no multiple representations of the same bone are found, and no clear evidence of individuals of different ages is recognizable. Similarly, the poor representativeness of the bone assemblage does not allow for the use of macro-morphological diagnostic criteria [52] nor the use of osteometric methods (e.g., [53]).

Concerning the age-at-death estimation, a few cranium fragments (n = 9) show portions of cranial sutures. However, they are characterized by profound thermal-related changes in shape. Amongst the few diagnostic traits, the preserved maxillary bone portion exhibits a complete fusion of the palatal suture [52], thus suggesting the presence of an adult individual. Despite the previous gross morphological assessment by Becker [44,45], no sub-adult skeletal diagnostic features (e.g., incomplete epiphyseal fusion, teeth not yet fully formed and/or erupted) [54,55] were found within the assemblage.

### The faunal sub-sample

**Fig 6:**
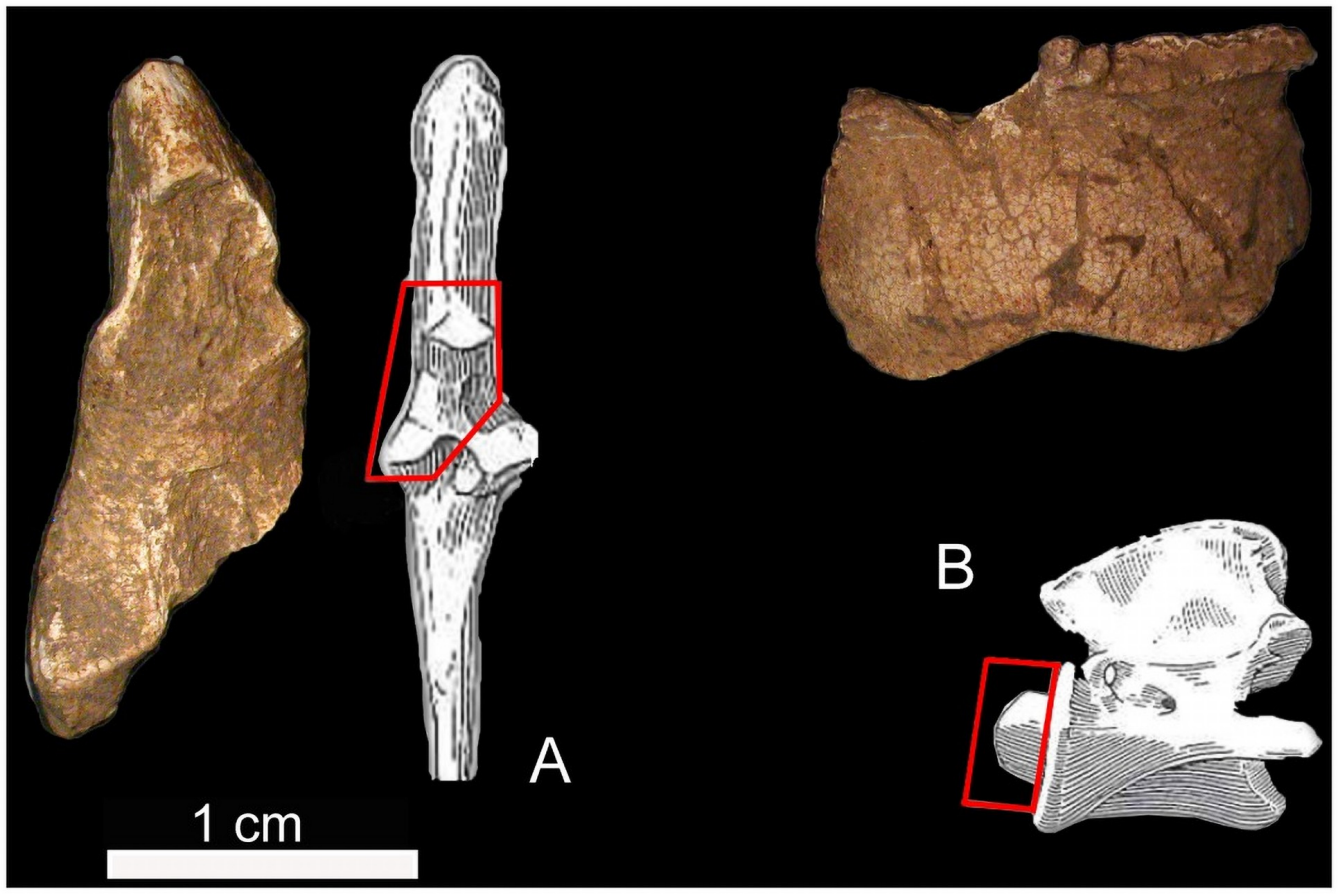
Faunal remains from the Tomb of Nestor’s Cup. cf. *Ovis aries*: Articular fragment of the left ulna (A) and odontoid process of the axis (B) with the fragments’ position on the anatomical reference element.

Faunal remains identified by gross morphological analysis consist of 45 burned fragments of varying size (from <1 cm to ~ 5 cm), weighing overall 39.2 g out of 218.4 g (18% of the total weight sample). Two out of 45 fragments were recognized as *Ovis/Capra*, probably *Ovis aries* [56,57]. They include an axis fragment and a proximal portion of the ulna (Fig 6). Furthermore, a tibia shaft fragment with foramen for its morphological features and size was tentatively attributed to *Canis familiaris*.

The thickness and morphology of a few elements suggest the presence of a bird. However, the absence of specific diagnostic features does not allow to be confident with the latter diagnosis for all the fragments. The most frequent parts are diaphysis fragments of undetermined long bones belonging to medium-sized mammals. The combustion degree recorded for the faunal remains is perfectly comparable to that documented on human features.

### Histological and histomorphometric results

Qualitative histological analysis was performed on 40 thin sections (n = 22 specimens from the human sub-set; n = 18 specimens from the faunal sub-set). The gross morphological assessment drove the sampling. The selected human bone fragments pertain to the humerus (18%), the femur (18%), or, more generally, to large-diameter long bones (64%). Overall, the selected faunal bone fragments pertain to long bones (94%) and possible metapodial bones (6%).

**Fig 7:**
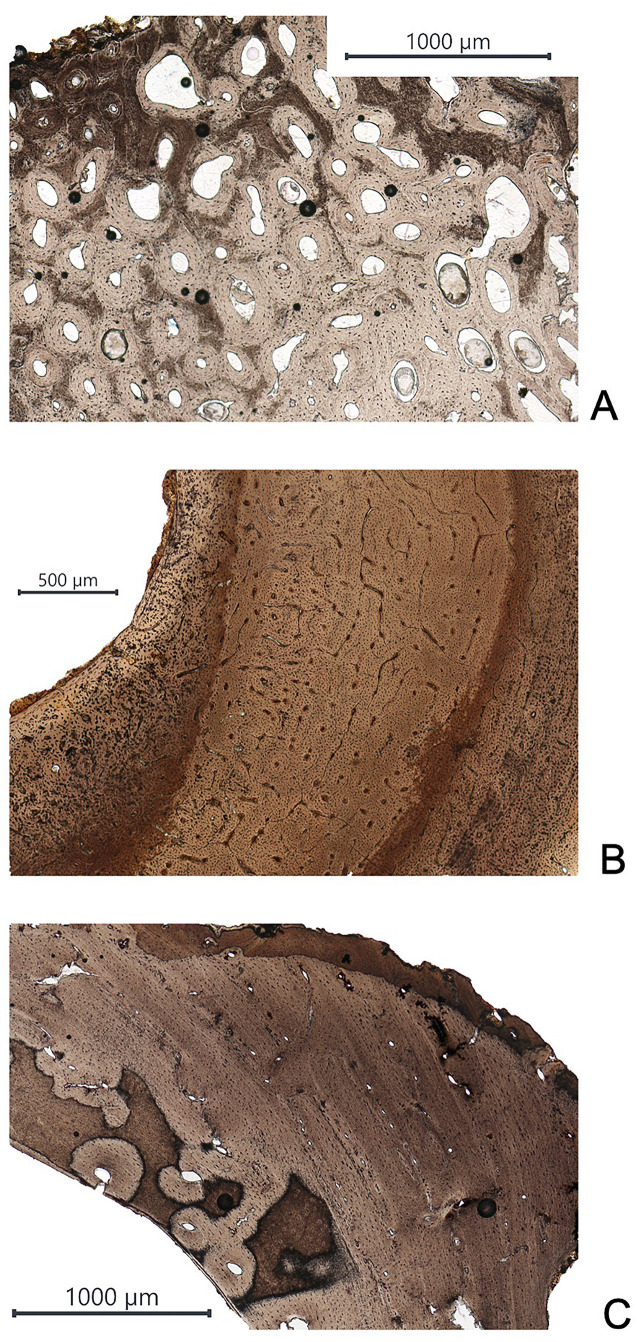
Comparison between thin sections in human and faunal specimens from the Tomb of Nestor’s Cup. (A) Thin section from the human specimen HIS-T(1); (B) thin section from the faunal specimen HIS-D(2); and (C) thin section from the faunal specimen HIS-H(2).

Table 3 (columns two and three) reports the gross morphological assessment and the blind test on the qualitative micro-features of the cortical bone in the human and faunal specimens (Fig 7).

**Table 3:** Gross morphology, histology, and histomorphometry comparison in the selected sample from the Tomb of Nestor’s Cup.

Specimen	Gross morphology	Histology	Histomorphometry
HIS-A(1)	Human	Human	Human
HIS-B(1)	Human	Human	Human
HIS-C(1)	Human	Human	Human
HIS-D(1)	Human	Human	Human
HIS-E(1)	Human	Human	--
HIS-F(1)	Human	Human	Human
HIS-G(1)	Human	Human	Human
HIS-H(1)	Human	Human	Human
HIS-I(1)	Human	Human	Human
HIS-J(1)	Human	Human	Human
HIS-K(1)	Human	Human	Faunal
HIS-L(1)	Human	Human	Human
HIS-M(1)	Human	Human	Faunal
HIS-N(1)	Human	Human	Faunal
HIS-O(1)	Human	Human	Human
HIS-P(1)	Human	Human	Human
HIS-Q(1)	Human	Human	Human
HIS-R(1)	Human	Human	Human
HIS-S(1)	Human	Human	Human
HIS-T(1)	Human	Human	Human
HIS-U(1)	Human	Human	Human
HIS-V(1)	Human	Human	Faunal
HIS-W(1)	Faunal?	Faunal?	--
HIS-X(1)	Faunal?	Faunal?	--
HIS-Y(1)	MSM	Faunal	--
HIS-Z(1)	MSM	Faunal?	Faunal
HIS-A(2)	MSM	Human	Human
HIS-B(2)	MSM	Faunal?	Human
HIS-C(2)	MSM	Faunal	--
HIS-D(2)	MSM	Faunal	--
HIS-E(2)	Aves?	Faunal?	Faunal
HIS-F(2)	Aves?	Faunal	--
HIS-G(2)	MSM	Faunal	--
HIS-H(2)	cf. *Canis familiaris*	Faunal	--
HIS-I(2)	Aves	Faunal	--
HIS-J(2)	Aves	Faunal	--
HIS-K(2)	MSM	Faunal	--
HIS-L(2)	MSM	Faunal	--
HIS-M(2)	MSM	Human	--
HIS-N(2)	MSM	Faunal	--

MSM (Medium-Sized Mammal).

Within the human sub-set, all specimens exhibit Haversian bone. The microscopic appearance is characterized by a slightly more significant presence of intact secondary than fragmentary secondary osteons (Fig 7a). Regarding the faunal sub-set, four types of micro-features of the bone structure are observed (Fig 7b and 7c): (i) primary vascular longitudinal bone with a band of primary osteons; (ii) primary vascular plexiform bone; (iii) combination of primary vascular longitudinal and irregular or dense Haversian bone; (iv) combination of primary vascular plexiform bone with irregular Haversian bone. The specimens HIS-A(2) and HIS-M(2) are morphologically recognized as medium-sized mammals. In contrast, the microscopic analysis of the cortical bone unveils the Haversian complex’s presence and no evidence of primary vascular longitudinal bone and primary vascular plexiform bone, thus non supporting the morphological diagnoses. Comparing the morphological assessment with the histological observations, it is possible to assume that the specimen HIS-G(2) may pertain to a medium-sized carnivore, possibly a dog, thus supporting the tentative attribution to *Canis familiaris* of the specimen HIS-H(2).

The quantitative histomorphometric analysis is performed on a sub-set of 21 thin sections from the human sub-sample (recognised as human both on the morphological and histological qualitative assessment) and four thin sections from the faunal sub-samples, characterised, the latter, by uncertain qualitative assessment. In the human sub-set, the specimen HIS-E(1) is excluded from the quantitative analysis due to its intense diagenetic alteration. Table 4 illustrates the quantitative histomorphometric analysis results, reporting the Secondary Osteon Area per mm^2^ (On.Ar.) and the Haversian Canal Area per mm^2^ (Hc.Ar.), when consistently measurable. Four specimens [HIS-K(1), HIS-M(1), HIS-N(1), HIS-V(1)], identified as human by morphology and qualitative histology, are below the threshold of 0.025 mm^2^ for the On.Ar. variable (see Methods). Consequently, they have been excluded from further analyses.

**Table 4:** The histomorphometric analysis of human and faunal specimens from the Tomb of Nestor’s Cup.

		Range			Range	
Specimen	On.Ar. [mm^2^]	Min [mm^2^]	Max [mm^2^]	Hc.Ar. [mm^2^]	Min [mm^2^]	Max [mm^2^]
HIS-A(1)	0.036	0.015	0.081	0.0045	0.0004	0.0117
HIS-B(1)	0.045	0.045	0.046	0.0026	0.0009	0.0051
HIS-C(1)	0.074	0.131	0.131	0.0121	0.0054	0.0249
HIS-D(1)	0.062	0.047	0.060	0.0079	0.0169	0.0169
HIS-F(1)	0.034	0.013	0.071	0.0036	0.0008	0.0118
HIS-G(1)	0.030	0.018	0.039	0.0027	0.0015	0.0037
HIS-H(1)	0.105	0.032	0.501	0.0112	0.0042	0.0257
HIS-I(1)	0.044	0.023	0.076	0.0061	0.0230	0.0760
HIS-J(1)	0.052	0.030	0.073	0.0024	0.0300	0.0730
HIS-K(1)	0.019	0.001	0.029	0.0009	0.0013	0.0290
HIS-L(1)	0.036	0.022	0.056	0.0025	0.0220	0.0560
HIS-M(1)	0.022	0.013	0.034	0.0009	0.0130	0.0340
HIS-N(1)	0.012	0.008	0.020	0.0008	0.0080	0.0200
HIS-O(1)	0.029	0.016	0.038	0.0030	0.0160	0.0380
HIS-P(1)	0.048	0.029	0.076	0.0059	0.0290	0.0760
HIS-Q(1)	0.031	0.010	0.068	0.0065	0.0100	0.0680
HIS-R(1)	0.030	0.014	0.046	0.0027	0.0140	0.0460
HIS-S(1)	0.043	0.015	0.070	0.0061	0.0150	0.0700
HIS-T(1)	0.056	0.032	0.102	0.0074	0.0320	0.1020
HIS-U(1)	0.034	0.010	0.049	0.0031	0.0100	0.0490
HIS-V(1)	0.022	0.012	0.035	0.0011	0.0002	0.0020
HIS-Z(1)*	0.025	0.015	0.034	0.0018	0.0016	0.0022
HIS-A(2)*	0.033	0.018	0.055	--	--	--
HIS-B(2)*	0.025	0.026	0.035	--	--	--
HIS-E(2)*	0.022	0.006	0.054	--	--	--

Secondary Osteon Area (On.Ar.) [mm^2^] and Haversian Canal Area (Hc.Ar.) [mm^2^].

* = faunal remains characterized by uncertain qualitative assessment.

#### Osteon population density in the human sub-sample

Specimens diagnosed as human by morphology, qualitative histology and histomorphometric analyses (n = 17) were analysed for Osteon Population Density (OPD) to evaluate possible heterogeneity in age at death. Results of the OPD analysis are reported in Table 5. The OPD values range between 5.96 and 19.65.

**Table 5:** Histomorphometric analysis of the Osteon Population Density (OPD) in human specimens from the Tomb of Nestor’s Cup.

Specimen	Bone	Ct.Ar.	No. Osteons	OPD
HIS-A(1)	femur	15.3	296	19.28
HIS-B(1)	humerus	11.1	218	19.65
HIS-C(1)	femur	2.0	40	19.55
HIS-D(1)	femur	13.5	189	14.03
HIS-F(1)	humerus	16.4	101	6.18
HIS-G(1)	humerus	18.6	137	7.35
HIS-H(1)	femur	46.1	676	14.68
HIS-I(1)	long bone	21.0	213	10.14
HIS-J(1)	long bone	24.3	192	7.90
HIS-L(1)	long bone	11.9	78	6.58
HIS-O(1)	long bone	5.1	33	6.45
HIS-P(1)	long bone	9.1	98	10.74
HIS-Q(1)	long bone	17.7	192	10.82
HIS-R(1)	long bone	24.6	159	6.48
HIS-S(1)	long bone	33.9	202	5.96
HIS-T(1)	long bone	48.3	521	10.78
HIS-U(1)	long bone	27.8	311	11.19

Cortical Area (Ct.Ar.) [mm^2^]; Osteon Population Density (OPD).

**Fig 8:**
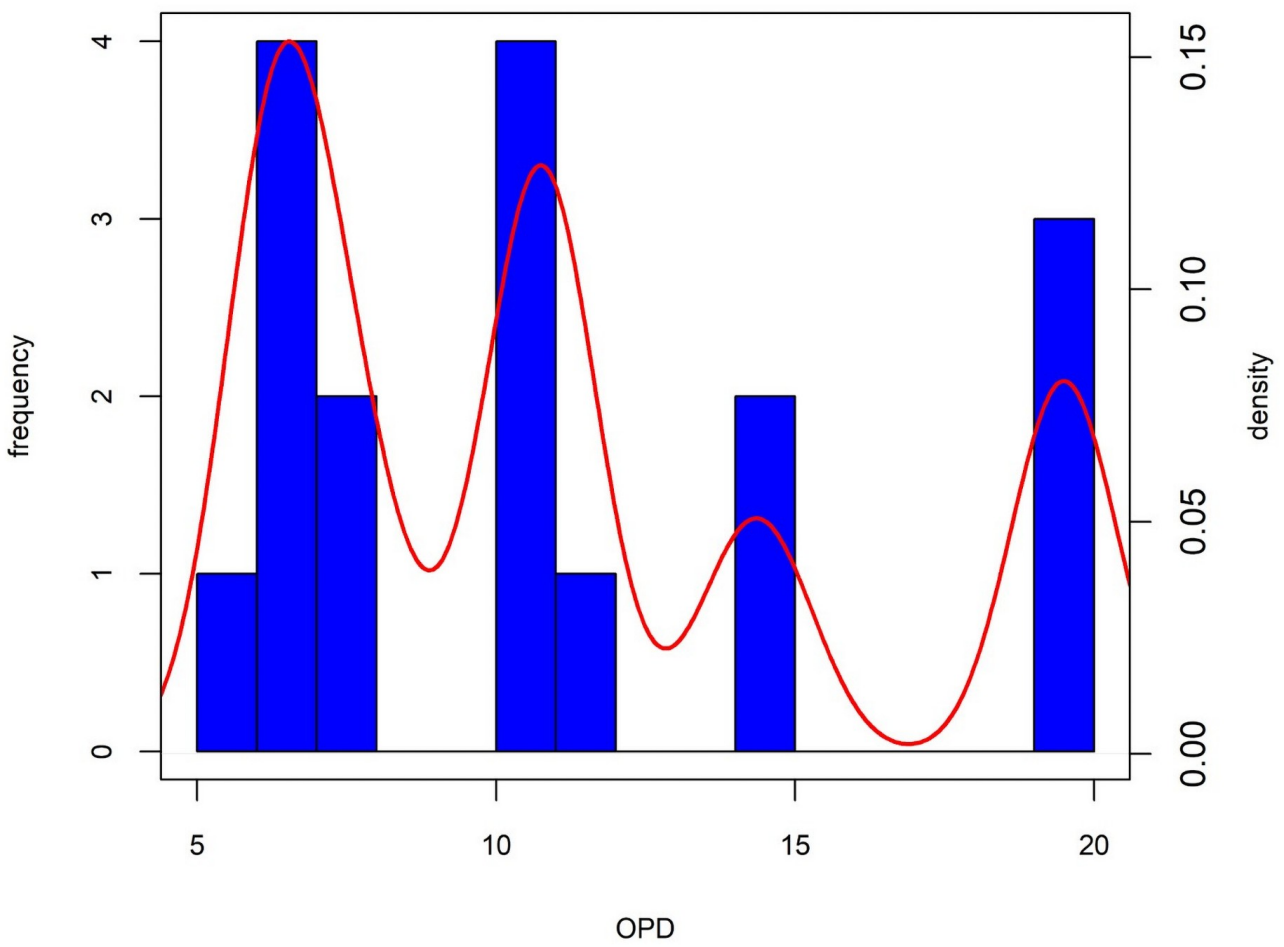
Histogram and density distribution plot of the logtransformed OPD values in the human bones from the Tomb of Nestor’s Cup. The bandwidth of the density estimate = 0.07.

The distribution of the logtransformed (as in [58]) OPD values is multimodal (Fig 8). The optimal number of clusters was determined using the fviz_nbclust routine of the factoextra R package (optimal number of clusters = 3) with the Gap statistic [59] used to calculate the goodness of clustering measure. Using a k-means algorithm the distribution of the logtransformed OPD was partitioned into three different OPD clusters: (i) [5.96; 7.9] (n = 7 specimens); (ii) [10.14; 11.19] (n = 5 specimens); (iii) [14.03; 19.65] (n = 5 specimens) (Fig 8). The means of the three OPD clusters are statistically different (Kruskall-Wallis rank sum test, chi-squared = 14.12, df = 2, p-value<0.01; analysis of variance test, df = 2.14, F = 104.6, p<0.0) and the Tukey’s Honest significant difference test indicates significant differences between the clusters.

## Discussion

The analysis of the macroscopic and microscopic bone structure of the cremated remains of Tomb 168 at Pithekoussai allowed the recognition, for the first time, of the commingled nature of the skeletal assemblage from the Tomb of Nestor’s Cup. Our results differ from previous morphological assessments of the cremated remains which identified the cremation deposit as the burial of a single individual aged between 10 and 14 years of age-at-death (see S2 Text in S1 File and S1 Fig) [44,45]. The most significant discrepancies concern: (i) the presence of faunal bones commingled with the human remains; (ii) the number of human individuals in the cremation deposit and their age-at-death.

The only animal confidently identified is *Ovis/Capra*, probably *Ovis aries*, while the occurrence of *Canis familiaris* is tentative on a morphological basis. The general class Aves is also represented. Animal remains in Cremation 168 are a novelty evidenced for the first time by the present study, but they do not represent an *unicum* within Pithekoussai necropolis. Portions of animals are attested in 45.31% in Late Geometric II cremations at the site. Single female cremations are usually associated only with *Ovis/Capra* and *Sus domesticus*. At the same time, males are accompanied by a broader range of species, such as *Ovis/Capra*, *Sus domesticus*, *Bos taurus*, and *Canis familiaris*. Lastly, Aves, *Ovis/Capra*, and *Sus domesticus* were found in double (male and female) cremations [5,60].

As far as the human cremated remains are concerned the Tomb 168 shows a substantial under-representation in weight when compared both to modern and archaeological cremation deposits [47,61–64]. However, the consistency of the cremated remains in Tomb 168 aligns well with the paucity of bones in single and double cremations during the Late Geometric II (720–690 BCE) at Pithekoussai (single female cremations median = 156 g; single male cremations median = 256 g; double cremation median = 233 g) [5,60]. The lack of elements of the axial skeleton (4% of the total weight) is evident when considering that this anatomical district is expected to be at least 30% in cremated remains from forensic contexts and 18% from archaeological ones [65]. Furthermore, the paucity of specific anatomical districts, i.e., facial bones, axis, pelvis, and hand and foot bones compared to the more represented cranium and long bones, might be a sign of a selective collection of the burnt remains, where cranial and long bones had been favoured over other districts. Besides the incomplete recovery of the cremated remains from the pyre, the absence of non-perishable urns and the alteration of the Tomb by subsequent depositions might have played a vital role in affecting the Cremation 168 bone assemblage.

As for the other Pithekoussai’s cremation deposits, the analysis of the heat-induced bone modifications in Tomb 168 shows no significant differences between the burning patterns of human and faunal remains. Animals, or portions of them, were burnt together with or in the same way as the human corpses, and they may represent food offerings for the deceased(s) or companions in the journey to the afterlife [66].

The qualitative histological blind test for taxon recognition of the bone fragments identifies as human 100% of the bones classified by gross morphology. For the faunal sub-set, gross morphology agrees with the qualitative histological assessment in 89% of cases. Similarly, four specimens, recognised as human by morphology and qualitative histology, have been identified as faunal by histomorphometric analysis. We would stress that histology basically looks at the type of features seen in cortical bone (namely, Haversian system, plexiform bone, etc. see Methods), while histomorphometrics relies on measurements of specific traits of bone microstructures (see S1 Table). Hence, the reason for different groupings for a very few specimens is based on how the features could be observed (for example, fragments with plexiform bone did not require osteon measurements to suggest human vs non-human identification).

**Fig 9:**
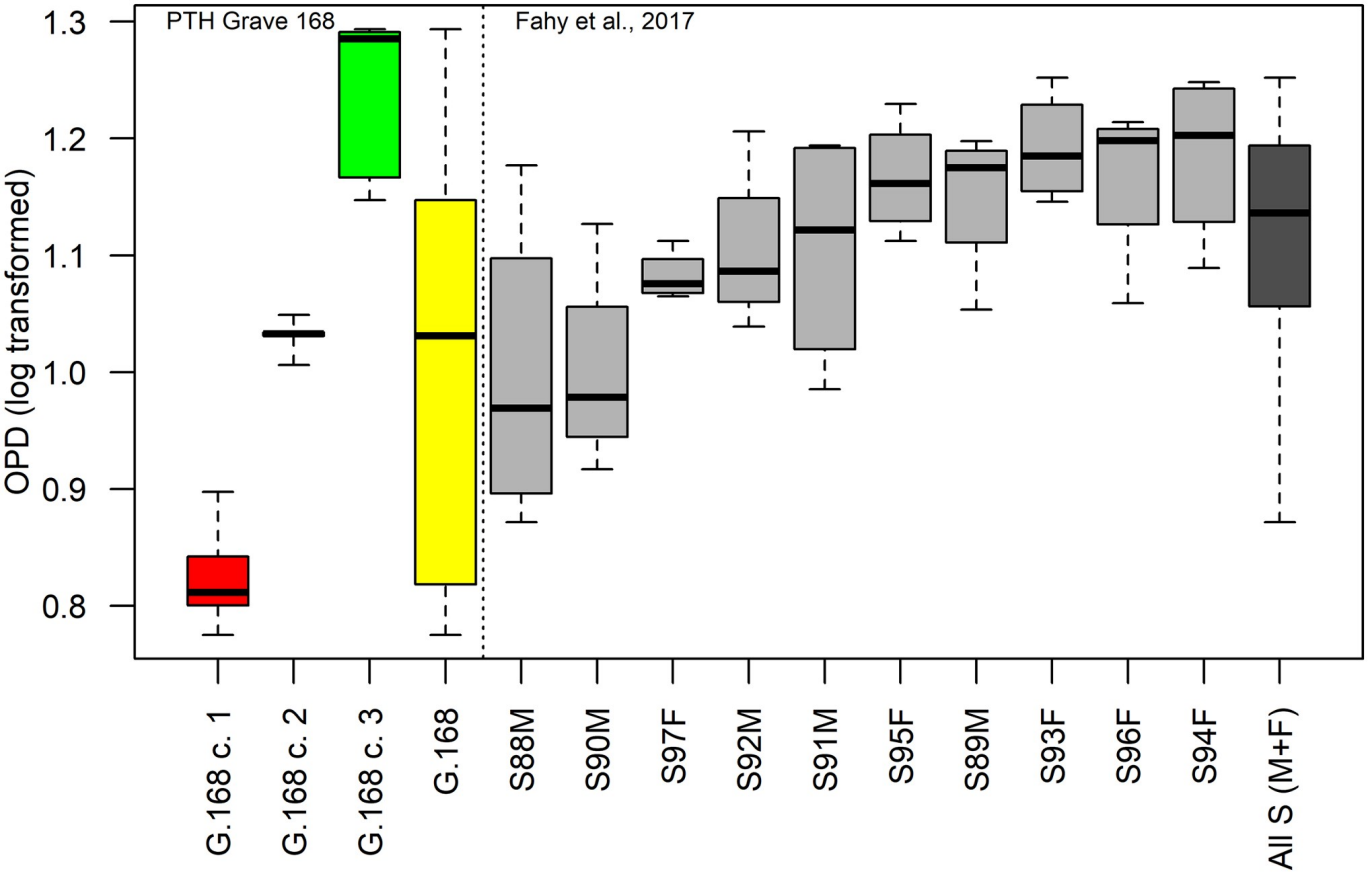
Comparison of the logtransformed OPD values from the human cremated samples of the Tomb of Nestor’s Cup and the logtransformed OPD individual values in long bones following [58]. The box and whisker plots of the Tomb 168 lontransformed OPD values by the three clusters (cluster 1, *red*; cluster 2, *black*; cluster 3, *green*) and the whole range of variation (*yellow*) compared to the logstransformed OPD published data (*grey*) from 10 adults (5 males and 5 females). The whisker plot shows a narrower range of variation amongst the published subset comparing to OPD values from Tomb 168.

Results of the human OPD values show that humeral specimens are distributed in two out of three OPD clusters: HIS-F(1) and HIS-G(1), fall in the first OPD cluster, whereas the humeral specimen HIS-B(1), with the highest OPD value, falls in the third OPD cluster. To support this conclusion, we compared our results with a histologically examined unburned bone collection of 10 early medieval individuals from the St Gregory’s cemetery in Canterbury, England, that considers different anatomical districts from single skeletal units [58]. The ages-at-death of the comparative series range between 25 and 35 years, and 5 males and 5 females are represented. Fig 9 shows the box and whiskers plot of the Tomb 168 logtransformed OPD values compared with the homologous values from [58], using only long bones OPD data (viz., humerus, radius, femur and tibia). The whole range of variation of the comparative sample is narrower than the range of Tomb 168 remains.

The medians of cluster one and cluster three of Tomb 168 lies outside the range of variation of the comparative sample. Therefore, it is highly probable that the OPD clusters point at separate individuals and not the micro-anatomical variations of a single one, although the thin sections were obtained from diverse long bones (e.g., humerus, ulna and radius, femur, tibia and fibula). Given the homogeneity in external appearance (colour, texture, etc), a differential thermal alteration is probably to be ruled out as a causal factor for the OPD values variability. Therefore, the cremated remains of Tomb 168 probably represent at least three individuals.

Regarding the age-at-death estimate, even though the correlation between an increasing density of OPD values and advancing age is well documented in adult and juvenile bone tissue [38,67,68], the cremated nature of the osteological record does not allow the reliable use of any age-at-death prediction model or reference values reported in the relevant literature for unburned remains [58,69,70]. However, the medians’ distribution of the three OPD clusters in Fig 9, suggest that the cremated remains represent three individuals ranging from a younger (first cluster) to an older one (third cluster). Overall, the OPD values possibly exclude the presence of children in the Tomb 168 bone assemblage. Therefore, the OPD values analysis which presents at least three human individuals might support the initial hypothesis by Buchner, who interpreted Cremation 168 as the outcome of three distinct depositions [13]. Remarkably, no other cremation deposits with more than two individuals are attested at Pithekoussai.

## Conclusion

Since the discovery and the early description of the Tomb of Nestor’s Cup, there has been a considerable debate on the nature of the osteological remains and whom they might relate to. Much of this debate has been focused on the unique material culture regarding the significant association of the inscribed Cup with a juvenile. In this study, morphological and histomorphometric analyses provide evidence that some of the burnt skeletal remains from the Tomb of Nestor’s Cup are not human, testifying to the presence of faunal remains commingled with the human ones. Such evidence matches the use of faunal portions during the cremation ritual, already observed within Pithekoussai’s necropolis. Our results point to the presence of at least three human individuals of different ages buried with Nestor’s Cup, possibly reinforcing Buchner’s initial reconstruction of the three distinct cremations. Even if it is not possible to estimate these three individuals’ age-at-death, none of them seems to pertain to a child. Henceforth, the association of the prestigious inscribed *kotyle* with a single juvenile individual does not find here confirmation. This study significantly contributes and takes forward the debate on the interpretation of the complex and unique context of the Tomb of Nestor’s Cup, opening to new questions about the nature of the burial(s) and the meaning of the Cup and its inscription buried there.

## Methods

Gross morphology and osteometric analyses were performed on cremated remains to (i) identify the possible presence of faunal remains amongst human fragments [71]; (ii) determine the MNI [72]; (iii) estimate the age-at-death and possibly the sex of the individual(s) [52,53]. Fragments found to conjoin were reconstructed using water-soluble glue, helping the gross interpretation, and facilitating the elements’ taxonomic assessment and recognizing diagnostic skeletal features [73]. Bone weight and representativeness were recorded for the main anatomical districts [47,61,74,75]. Taxonomic assessment of faunal remains was performed by comparing the faunal reference collection stored at the Bioarchaeology Service, Museum of Civilization in Rome. Given the high degree of fragmentation of the specimens, when a taxonomic attribution to animal species was not possible, more general categories were employed. For example, Medium-Sized Mammals (MSM) would include sheep, goat, pig, dog and other animals of similar size. Non-human bone remains were distinguished based on morphological features when it was possible to identify skeletal element and species, while for the diaphyses the assessment was based on size, thickness, and cross-section of the shaft fragments in addition to the gross evaluation of bone density, the porosity of surfaces, as well as presence/absence, distribution, and shape of trabeculae [56,57]. Observations and descriptions of heat-induced changes on the bones were performed, recording variations in colour, strength, porosity, dimension, weight loss and heat-induced fractures [47,50]. The observations were made using a 10-power hand lens under proper artificial light. Where the morphologies were ambiguous, fragments were further examined under a stereomicroscope (Nikon SMZ1000).

Following gross morphology and osteometric assessments, 40 specimens out of 195 bone fragments were histologically examined. The 40 specimens from Tomb 168 were labelled with a sequential alpha-numeric identification [HIST-A(1); B(1); C(1), etc.] before the cutting and analysing processes to guarantee a blind histological assessment. No taxonomic or individual indications were reported on the specimens. Four independent observers performed micro-feature analysis. The purpose of this assessment was to provide specific data regarding the nature of the bone fragments, including (i) histomorphology and histomorphometric differences related to human and non-human variations; (ii) micro-morphological and histomorphometric differences related to age-specific variation within the human sub-sample. The rationale for doing this assessment was inspired by Stout and Gelher [76], where it is successfully highlighted that histological features are helpful in accurately sorting out commingled human remains. By doing so, several histomorphometric features were recorded. These included Secondary Osteon Area per mm^2^ (On.Ar.) and Haversian Canal Area per mm^2^ (Hc.Ar.) (see S1 Table), as well as recording the features specific to non-human bone micro-anatomy (e.g., primary vascular plexiform bone and/or osteon bands) [20,23,24,26,28,31,70,77]. The On.Ar and Hc.Ar. nomenclature follows Parfitt *et al*. [78]. To distinguish faunal versus human remains, a threshold of 0.025 mm^2^ of On.Ar. has been adopted [30,34,42,79,80]. All the specimens below this threshold are considered faunal in origin [30,32,33]. Due to the highly fragmentary state of this skeletal assemblage and the cremated nature of remains, the three analytical methods used for the study of the cremation deposit (namely, morphology, histology, and histomorphometry) are affected by an error rate, although minimal. Consequently, only those fragments diagnosed as human by all three methods were used for the estimation of the minimum number of individuals in Tomb 168, through the OPD estimation.

The histological and histomorphometric analyses were performed at the Bioarchaeology Service, Museum of Civilization in Rome. Thin cortical bone sections were obtained using the method proposed by Nava *et al*. [81] to prepare thin dental sections, with some modification (S3 Text in S1 File). Microstructural data were collected using a transmitted light microscope (Olympus BX 60) under polarised light with a magnification of 40x. Overlapping pictures were taken for each specimen (typically 20–30 images at 40x) using a camera (Nikon DSFI3) paired with the optical microscope, later assembled in a single micrograph through the software ICE 2.0 –Image Composite Editor (Microsoft Research Computational Photography Group). Histomorphometric measurements were taken from calibrated photo-mosaic using Fiji image analysis software [82]. The criteria recommended by Nor *et al*. [28] were used for osteon counting. All statistical analyses and graphs were done with the R language and environment for statistical computing [83] (ver 4.1.0), the package ***‘***BlandAltmanLeh***’*** [84], and the ‘factoextra’ package [85].

The cremated remains from Tomb 168 (Pithekoussai, Buchner’s excavation 1952–1961) included in this research, as well as the entire osteological collection from Pithekoussai necropolis are temporarily stored at the Bioarchaeology Service of the Museum of Civilizations in Rome (Ministero della Cultura). All necessary permits were obtained for the described study, which complied with all relevant regulations. All necessary permits were granted by the Soprintendenza Archeologia Belle Arti e Paesaggio per l’Area metropolitana di Napoli (Ministero della Cultura), which is legally responsible for the entire osteological collection from Pithekoussai necropolis.

## Supporting information

S1 Fig Bland-Altman plot of the differences in the individual cremation deposits’ weight between Becker study [6,7] and the present research. The graph shows on the Y-axis the difference between the two paired measurements (Becker’s weights -present study weights), and the X-axis represents the average of these measurements; the dashed lines report the mean of the differences and the ±2 s.d. interval.
(TIF)

S1 Table Comparison of the non-human micro-anatomy mean measurements from previous studies. (DOCX)

S1 File (DOCX)
