# Supplementary figures and images for: Who was buried with Nestor’s Cup? Macroscopic and microscopic analyses of the cremated remains from Tomb 168 (second half of the 8th century BCE, Pithekoussai, Ischia Island, Italy)
Source: PLoS One. 2021 Oct 6;16(10):e0257368. doi: 10.1371/journal.pone.0257368 (PMC8494320; doi:10.1371/journal.pone.0257368)

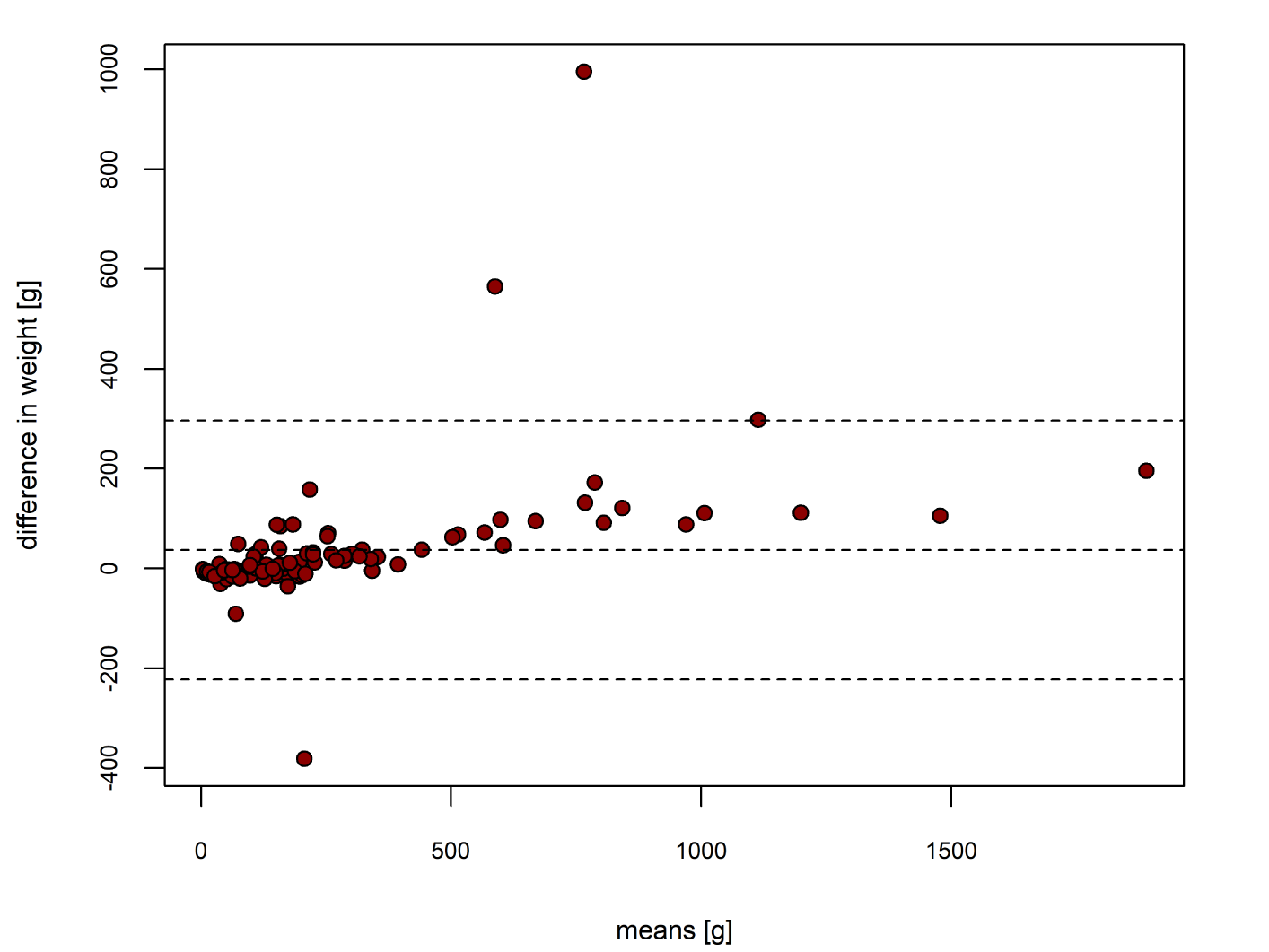

Supplement: S1 Fig — The graph shows on the Y-axis the difference between the two paired measurements (Becker’s weights -present study weights), and the X-axis represents the average of these measurements; the dashed lines report the mean of the differences and the ±2 s.d. interval. (TIF) [file pone.0257368.s001.tif]
