# Supplementary material for: Who was buried with Nestor’s Cup? Macroscopic and microscopic analyses of the cremated remains from Tomb 168 (second half of the 8th century BCE, Pithekoussai, Ischia Island, Italy)
Source: PLoS One. 2021 Oct 6;16(10):e0257368. doi: 10.1371/journal.pone.0257368 (PMC8494320; doi:10.1371/journal.pone.0257368)
Supplement: S1 Table — (DOCX) [file pone.0257368.s002.docx]

**S1 Table. Comparison of the non-human micro-anatomy mean measurements from previous studies.**

|  | On.Ar. | Hc.Ar. |
| --- | --- | --- |
| Dog | 0.01862^1^ | 0.000575^1^ |
| Dog | 0.01403^3^ | 0.000694^3^ |
| Dog | 0.0103^4^ | 0.000392^4^ |
| Dog | 0.01404^5^ | -- |
| Pig | 0.0113^4^ | 0.000602^4^ |
| Deer | 0.00741^3^ | 0.000409^3^ |
| Deer | 0.0113^4^ | 0.000245^4^ |
| Sheep | 0.01645^3^ | 0.000574^3^ |
| Human | 0.03905^1^ | 0.002382^1^ |
| Human | 0.05364^2^ | 0.00223^2^ |
| Human | 0.04412^3^ | 0.002877^3^ |

^1^Jowsey 1966 [1]; ^2^Horni 2002 [2]; ^3^Urbanová and Novotný, 2004 [3]; ^4^Morris 2007 [4]; ^5^Sawada *et al.* 2014 [5].

**Reference**

1. Jowsey J. Studies of Haversian systems in man and some animals. J Anat. 1966; 100(4):857-864.

2. Horni H. The forensic application of comparative mammalian bone histology. M. SC. Thesis, Texan tech University. 2002. Available from: <https://ttuir.tdl.org/bitstream/handle/2346/10476/31295017084319.pdf?sequence=1>

3. Urbanová P, Novotný V. Distinguishing between human and non-human bones: histometric method for forensic anthropology. Anthropologie. 2004; XLII/2:175-183.

4. Morris ZH. Quantitative and Spatial Analysis of the Microscopic Bone Structures of Deer (Odocoileus virgilianus), Dog (Canis familiaris) and Pig (Sus scrofa domesticus). MS.C. Thesis. Louisiana State University and Agricultural and Mechanical College. Baton Rouge. LA. 2007.

5. Sawada J, Nara T, Fukui J, Dodo Y, Hirata K. Histomorphological species identification of tiny bone fragments from a Paleolithic site in the Northern Japanese Archipelago. J Archaeol Sci. 2014; 46:270-280.
