# Supplementary material for: Who was buried with Nestor’s Cup? Macroscopic and microscopic analyses of the cremated remains from Tomb 168 (second half of the 8th century BCE, Pithekoussai, Ischia Island, Italy)
Source: PLoS One. 2021 Oct 6;16(10):e0257368. doi: 10.1371/journal.pone.0257368 (PMC8494320; doi:10.1371/journal.pone.0257368)
Supplement: S1 File — (DOCX) [file pone.0257368.s003.docx]

**Supporting Information**

**S1 Text. Chronological assessment of the archaeological context**

The chronological assessment of Cremation 168 was of utmost importance in the studies that followed the first publication of this Tomb. The dating of Pithekoussai graves is a cornerstone for the relative chronology of the transitional phases from the Iron Age to the Orientalizing period (8^th^-7^th^ century BCE) in the western Mediterranean.

Initially, Buchner proposed a date in the third quarter of the eighth century BC for the inscribed *kotyle*, and ***‘***well within the eighth century BC***’*** for the grave goods as a whole [1]. In 1987, Neeft challenged this chronology [2] based on the Protocorinthian globular *aryballoi* style, which he considered to be later in the typological sequence than those from Tomb 325. This latter being dated at 718/717– 712 BCE for the scarab with the cartouche of pharaoh Bocchoris. The lower date was believed to be further supported by the date of two local conical lekythoi found in the ***'***sherd layer***'***, which presumably set a *terminus post quem* for Tomb 168 in Late Geometric II, between 720 and 690 BC in the chronology proposed by Coldstream [3]. Subsequently, V. Nizzo stepped into this debate, reinterpreting the overall necropolis framework and proposing a reassessment of the stratigraphic relations. He also stressed the chronological differences between the materials from Tomb 168, supporting Buchner's initial statement that this Tomb comprised at least two burials [4, 5]. According to this scholar, of the *two* burials, the earlier one (Tomb 168A), including Nestor’s Cup, would retain its position at the beginning of Late Geometric II, while the more recent one (Tomb 168B) would be closer in date to Tomb 325. The four craters would remain outside of the two burial assemblages, being ascribed instead to the ***‘***layer of sherds mixed with brown earth***’*** that extended in the vast space between Tombs 168 and 180. This interpretation has the limit – recognized by Nizzo himself – of being an *ex-post* reconstruction based on assumptions that can no longer be tested. Nevertheless, the hermeneutic potential of this hypothesis can hardly be denied.

**S2 Text. Discrepancies in bone assemblage weights**

Comparing the previous assessment of Tomb 168 skeletal assemblage and our results, the weights in grams are different. Overall, the weight of the cremated remains here recorded is of 218.4 g, whereas Becker reported 289 g [6, 7]. Discrepancies in weights between Becker's initial measurements and the present status of the cremated remains are also recorded in other Pithekoussai cremations, as shown by the Bland-Altman graph in S1 Fig; 55.4% of the cremated remains are greater in weight in the Becker's record. The mean of the absolute difference between the two sets of weights is 54.5 g (n = 101, s.d. = 122.7 g, range = 0.1 – 995.0 g, median = 15.9 g). A possible explanation for this difference is the loss of osteological material, especially minute fragments, resulting from previous examinations and/or storage [5]. However, this hypothesis does not account for Becker's weights lighter than the present study measurements (45.6% of the cases).

**S1 Fig. Bland-Altman plot of the differences in the individual cremation deposits’ weight between Becker study [6, 7] and the present research.** The graph shows on the Y-axis the difference between the two paired measurements (Becker's weights -present study weights), and the X-axis represents the average of these measurements; the dashed lines report the mean of the differences and the ±2 s.d. interval.

**S3 Text. Methods of histomorphometric analysis of the cortical bone: sampling and thin sections procedures**

The specimen under analysis was fixed with the proper orientation in a mould, specifically chosen to fit inside the microtome holder, using a small amount of light-curing composite resin (Charisma, Heraus Kulzer). The composite was polymerized using a cure light (MectronBluelight) for 60 seconds. The specimen was then completely embedded in epoxy resin, which was obtained by mixing the resin with the hardener (Epo-Fix, Buehler Ltd) in the ratio 50g resin for 10g hardener. The mould was inserted in a vacuum chamber for 15 minutes to remove air bubbles and then left curing for 24 hours at room temperature. Blocket with the embedded bone was sectioned using a Diamond Blade Microtome (Leica 1600, Leica AG or BuhelerIsomet low speed). A first cut was done to remove the outer part of the resin block and expose the bone fragment's inner surface. The holder was repositioned 600 μm under the exposed surface to obtain a ~300 μm thick section. A microscope slide previously treated with liquid silane (3 M RelyX Ceramic Primer) was attached to the exposed surface before sectioning using a light cure adhesive (3 M Scotchbond Multi-Purpose Adhesive) polymerized for 60 seconds. A thin section averaging 300 μm was cut with the microtome arm advancing at medium-low speed. The section was reduced to a thickness of ~100 μm. This step was performed employing a motorized grinder (Minimet 1000 Automatic Polishing Machine, Buehler) with the water-resistant abrasive paper of two different grits (400 and 1200, Carbimet, Buehler Ltd). The section was polished using the motorized grinder with a micro-tissue (Buehler Ltd) and diamond paste with oneμm size (DB-Suspension, M, Struers) least 15 minutes to remove the grooves made by the blade and the grinding procedure. Micrographs of the samples were obtained using a transmitted light microscope (Olympus BX 60) under polarised light with a magnification of 40x. Overlapping pictures were taken for each specimen (typically 20-30 images at 40x) using a camera (NIKON mod. DS-Fi3) attached to the microscope and then assembled in a single image using dedicated software (Fiji-plugin MosaicJ, [8] and ICE 2.0 – Image Composite Editor (Microsoft Research Computational Photography Group).

**References Supporting Information**

1. Buchner G, Russo CF. La coppa di Nestore e un’iscrizione metrica da Pitecusa dell’VIII secolo av.Cr: Rendiconti lincei. Scienze morali, storiche e filosofiche. Roma: Accademia Nazionale dei Lincei; 1955.

2. Neeft CW, Strietman G. Protocorinthian subgeometric aryballoi. Amsterdam:Allard Pierson Museum; 1987.

3. Coldstream J. Greek Geometric Pottery: a Survey of Ten Local Styles and their Chronology. . 2nd ed. Exter: Bristol Phoenix Press; 2008.

4. Nizzo V. Cronologia versus Archeologia. L’ambiguo scorrere del tempo alle soglie della ‘colonizzazione’: i casi di Cuma e Pithekoussai. In: Donnellan L, Nizzo V, Burgers G, editors. Contexts of early Colonization Contextualizing Early Colonization Archaeology, Sources, Chronology and Interpretative Models between Italy and Mediterranean (Rome 2012). Rome: Papers of the Royal Netherlands Institute in Rome; 2016. p. 49-72.

5. Nizzo V. Ritorno ad Ischia: dalla stratigrafia della necropoli di Pithekoussai alla tipologia dei materiali. Naples: Publications du Centre Jean Bérard; 2007.

6. Becker MJ. Human skeletal remains from the pre-colonial Greek emporium of Pithekoussai on Ischia: culture contact in the early VIII to the II century BC. In: Neil C, editor. Settlement and economy in Italy, 1500 BC-AD 1500. Papers of the 50^th^ Conference of Italian Archaeology. Oxford: OXBOW Books; 1995. pp. 273-281.

7. Becker MJ. Human skeletons from the Greek emporium of Pithekoussai on Ischia (Na): culture contact and biological change in Italy after the 8th century BC. In: Morter J, Tykot RH, Robb JE, editors. Social Dynamics of the Prehistoric Central Mediterranean. London: Accordia Research Institute, University of London; 1999. pp. 217-225.

8. Thevenaz P, Unser M. User-friendly semiautomated assembly of accurate image mosaics in microscopy. Microsc Res Tech. 2007; 70(2):135-46.
